# Decoding negative affect personality trait from patterns of brain activation to threat stimuli

**DOI:** 10.1016/j.neuroimage.2015.12.050

**Published:** 2017-01-15

**Authors:** Orlando Fernandes Jr, Liana C.L. Portugal, Rita de Cássia S. Alves, Tiago Arruda-Sanchez, Anil Rao, Eliane Volchan, Mirtes Pereira, Letícia Oliveira, Janaina Mourao-Miranda

**Affiliations:** a Department of Physiology and Pharmacology, Laboratory of Neurophysiology of Behaviour, Biomedical Institute, Federal Fluminense University, Niterói, RJ, Brazil; b Department of Radiology, Faculty of Medicine, Clementino Fraga Filho University Hospital, Federal University of Rio de Janeiro, Rio de Janeiro, RJ, Brazil; c Laboratory of Neurobiology II, Institute of Biophysics Carlos Chagas Filho, Federal University of Rio de Janeiro, Rio de Janeiro, RJ, Brazil; d Department of Computer Science, Centre for Computational Statistics and Machine Learning, University College London, London, UK; e Max Planck University College London Centre for Computational Psychiatry and Ageing Research, University College London, London, UK

**Keywords:** Negative affect trait, Threat stimuli, Functional magnetic resonance imaging, Pattern recognition analyses, Multi-kernel learning, Decode

## Abstract

**Introduction:**

Pattern recognition analysis (PRA) applied to functional magnetic resonance imaging (fMRI) has been used to decode cognitive processes and identify possible biomarkers for mental illness. In the present study, we investigated whether the positive affect (PA) or negative affect (NA) personality traits could be decoded from patterns of brain activation in response to a human threat using a healthy sample.

**Methods:**

fMRI data from 34 volunteers (15 women) were acquired during a simple motor task while the volunteers viewed a set of threat stimuli that were directed either toward them or away from them and matched neutral pictures. For each participant, contrast images from a General Linear Model (GLM) between the threat versus neutral stimuli defined the spatial patterns used as input to the regression model. We applied a multiple kernel learning (MKL) regression combining information from different brain regions hierarchically in a whole brain model to decode the NA and PA from patterns of brain activation in response to threat stimuli.

**Results:**

The MKL model was able to decode NA but not PA from the contrast images between threat stimuli directed away versus neutral with a significance above chance. The correlation and the mean squared error (MSE) between predicted and actual NA were 0.52 (p-value = 0.01) and 24.43 (p-value = 0.01), respectively. The MKL pattern regression model identified a network with 37 regions that contributed to the predictions. Some of the regions were related to perception (e.g., occipital and temporal regions) while others were related to emotional evaluation (e.g., caudate and prefrontal regions).

**Conclusion:**

These results suggest that there was an interaction between the individuals' NA and the brain response to the threat stimuli directed away, which enabled the MKL model to decode NA from the brain patterns. To our knowledge, this is the first evidence that PRA can be used to decode a personality trait from patterns of brain activation during emotional contexts.

## Highlights

• Negative affect personality trait could be accurately decoded from patterns of brain activation to threat stimuli.
• The multiple kernel learning (MKL) approach showed the contribution of different brain areas to the predictions.
• Regions related to perception and emotional evaluation contributed to predicting the individuals' Negative Affect (NA).
• Our results suggest that the NA trait plays an important role in the brain response to threat stimuli.

## Introduction

A personality trait is composed of a set of emotional qualities that characterize and define each individual. Emotional experience has been described in two dominant dimensions, namely, negative affect (NA) and positive affect (PA) (Watson and Clark, 1992). The negative affect dimension refers to the extent to which a person feels a negative mood, including anger, nervousness, fear, guilt, and sadness (Clark and Watson, 1991). The NA trait is linked to poor self-esteem, pessimism, and a propensity to make somatic complaints (Clark et al., 1994, Watson and Clark, 1984, Watson and Pennebaker, 1989). Similarly, the positive affect dimension reflects positive mood states, including joyful, interested, excited and alert (Watson and Clark, 1992). Studies have found that negative affect and positive affect are related to neuroticism and extraversion, respectively (Watson and Clark, 1992, Watson and Naragon-Gainey, 2014, Watson et al., 2005). For example, a meta-analysis conducted by Kotov et al. (2010) showed that anxiety, depression, substance use disorder (SUD) and posttraumatic stress disorder (PTSD) are strongly correlated with neuroticism. Moreover, extraversion was inversely associated with depression and social phobia and positively associated with bipolar disorder (Watson and Naragon-Gainey, 2014). Neuroticism and extroversion are some of the “Big Five” basic dimensions of affect (Watson and Clark, 1992) and are strongly linked to psychopathology and mental disorders (Kotov et al., 2010, Watson and Naragon-Gainey, 2014, Watson et al., 2005). The “Big Five” dimensions refer to a model that describes human personality using 5 factors or dimensions, which include traits of extraversion, neuroticism, agreeableness, conscientiousness, and openness/intellect (Costa and McCrae, 1992, DeYoung, 2010, Markon et al., 2005, Watson et al., 1994).

Individual variability can influence brain responses to specific stimuli, particularly to emotional ones (Calder et al., 2011, Canli et al., 2002, Sanchez et al., 2015). In fact, with respect to emotional stimuli, individual differences in responses are the rule rather than the exception (Eugène et al., 2003, Hamann and Canli, 2004). For instance, the amygdala response to happy faces was not statistically significant in a group analysis, but the emotional effect became significant when the personality trait of extraversion was taken into account (Canli, 2004). In the same vein, trait anxiety shows a positive relationship with the amygdala response to angry and fearful faces (Ewbank et al., 2009). Considering that emotional brain responses vary according to personality traits, a challenging question is whether pattern recognition methods can decode an individual's personality trait from his or her pattern of brain activation to emotional stimuli.

Pattern recognition methods applied to fMRI have made it possible to decode sensorial and cognitive states solely from patterns of brain activation. Examples of these applications include decoding the category of an object (Behroozi and Daliri, 2014, Cox and Savoy, 2003, Shinkareva et al., 2008, Spiridon and Kanwisher, 2002), the orientation of a visual stimuli presented to the subject (Carlson, 2014, Haynes and Rees, 2005, Kamitani and Tong, 2005), mental states related to memory retrieval (Chadwick et al., 2010, Polyn et al., 2005), hidden intentions (Haynes et al., 2007), reading ability (He et al., 2013), age-related differences in connectivity networks (Vergun et al., 2013) and emotion expression (Harry et al., 2013). Pattern recognition approaches have also been used to identify relationships between patterns of brain structure or activity and continuous measures of behavior, i.e., as a *pattern regression analysis* (Cohen et al., 2011, Stonnington et al., 2010). Pattern regression analysis techniques are therefore very promising tools for identifying neurobiological measures that can predict or decode measures of individual variability such as personality traits, but its full potential is still unknown.

In the literature, few studies report the prediction of personality traits from patterns of brain activation or behavior measures. Recently, Kosinski et al. (2013) showed that accessible digital records of behavior (i.e., Facebook likes) can be used to automatically and accurately predict dimensions of personality traits. Nevertheless, the vast majority of studies of neuroticism and extraversion have focused on finding associations between the signal of individual regions and personality trait dimensions at the group level using univariate statistical analysis (Britton et al., 2007, Cohen et al., 2005, Deckersbach et al., 2006, Haas et al., 2006, Hooker et al., 2008, Paulus et al., 2003). For example, functional neuroimaging studies have suggested associations between neuroticism and neural activity in the anterior cingulate cortex (ACC) (Eisenberger et al., 2005), insula (Deckersbach et al., 2006), anterior fronto-median cortex (Britton et al., 2007), and amygdala (Hooker et al., 2008). Extraversion has been associated with neural activity in the striatum (Cohen et al., 2005), ACC (Canli, 2004), orbitofrontal cortex (Paulus et al., 2003), and amygdala (Canli et al., 2001). While studies such as these have employed univariate statistical analyses to identify associations between the signal within individual regions and dimensions of personality trait, the analysis methods used are limited in that they do not enable predictions at the individual subject level. Pattern recognition approaches such as the one used in the present study have the following 2 main advantages with respect to univariate analyses: (1) due to their multivariate properties they can achieve relatively greater sensitivity and are therefore able to detect subtle and spatially distributed effects; (2) they enable predictions for unseen subjects, providing information at the individual—rather than the group level. Here, we used a multiple kernel learning (MKL) approach, considering the whole brain multivariate pattern as a combination of regional patterns (Schrouff et al., 2014) to investigate the link between personality trait and patterns of brain activation to threat stimuli. The idea of the MKL approach is to hierarchically combine information from different brain regions into a whole brain model in which regions that carry more predictive information about the variable of interest (e.g., NA) will have a higher contribution to the model based on the region weights. The brain regions can then be ranked according to their contribution to the decision function, which facilitates the interpretation of the predictive model in terms of the contributions of different anatomical regions.

To best of our knowledge, the present study is the first to investigate whether NA and PA traits, as evaluated using the Positive and Negative Affective Schedule (PANAS, Watson et al., 1988), can be decoded from patterns of brain activation to threat stimuli in a healthy sample. In summary, the main aim of our study was to investigate whether pattern recognition methods could decode dimensional measures of personality traits from patterns of brain activation to threat. We focused on NA and PA traits because they are a relatively stable personality characteristics over time (Watson, 1988a, Watson, 1988b). As previously stated, NA can be considered as a risk factor for the development of mental health disorders, especially affective disorders, such as anxiety and depression (for review, see Ormel et al., 2004). On the other hand, PA trait could be an important component for determining human variability in threat perception and for modulating the emotional reactivity to threat stimuli (Oliveira et al., 2009, Sanchez et al., 2015). Thus, decoding NA and PA traits from patterns of brain activity could be potentially useful as a biomarker to identify individual risk for the development of psychiatric disorders.

## Methods

### Participants

Thirty-four undergraduate or graduate students without a history of neurological or psychiatric illness participated in the study (15 women; age range: 18–38 years). All of the participants had normal or corrected vision and gave written informed consent to participate in the study after the study was explained to them. The study was performed in accordance with the local Ethics Committee of the Federal Fluminense University, Brazil.

### Positive affect and negative affect traits

All of the participants completed personality traits assessment measures at the start of the experimental session, before entering the MRI scanner. The negative and positive affect traits were measured with the Positive and Negative Affect Schedule (PANAS) scale (Watson et al., 1988). The PANAS scale was designed to assess mood in general and can be used to assess mood at various time scales depending on the instructions. Possible time scales include moment, today, past few days, week, past few weeks, year, and general. In the present study, we assessed the mood state in general which relates to the participants' traits. The scale contains 20 words that describe different feelings and emotions. Ten words are related to positive moods (active, alert, attentive, determined, enthusiastic, excited, inspired, interested, proud and strong), and 10 words are related to negative moods (distressed, upset, hostile, irritable, scared, afraid, ashamed, guilty, nervous and jittery). Participants were asked to rate the degree to which they feel each emotion in general on a 5-point scale (1 = very slightly or not at all, 5 = extremely).

### Data acquisition

The data for this study were collected at the Department of Radiology at the Hospital Universitário Clementino Fraga Filho (Federal University of Rio de Janeiro, Brazil) on a 1.5-T Siemens (Magnetom Avanto) scanner. The fMRI runs were acquired on sequential ascending framework and using a gradient echo EPI single-shot sequence covering 25 axial slices (4 mm thick; 0.6 mm gap; TR/TE = 2000/40 ms; IST = 80 ms; FOV = 256 mm; matrix, 64 × 64; voxel dimensions, 4 × 4 × 4 mm). Head movements were restrained by foam padding. In each run, 198 functional volumes were acquired in four runs. In addition, a three-dimensional high-resolution T1-weighted anatomical image (TR/TE = 2730/3.27 ms, 128 slices, 0.6 mm gap, FOV = 250 mm, voxel dimensions 1.33 × 1 × 1.33 mm) was obtained at the beginning of the session for functional to anatomical image registration.

### Stimuli

Eighty-four pictures were used in this study. These 84 pictures consisted of threat and neutral stimuli (42 pictures each) that were split into 2 different sets. In the first set, the threat stimuli were pictures of a person holding a gun (threat stimuli). In the second set, the neutral stimuli were photos of a person holding a camera or a neutral object (neutral stimuli). In both sets, the guns or the neutral objects were either directed toward or away from the viewer (21 pictures in each). In summary, we have the following 4 pictures category in this experiment: (1) threat stimuli directed toward the viewer; (2) threat stimuli directed away from the viewer; (3) neutral stimuli directed toward the viewer; and (4) neutral stimuli directed away from the viewer. The pictures were matched in several properties to avoid confound effects that are unrelated to the effects of emotions on brain activity (Steinmetz et al., 2011). The properties controlled were ethnicity (balanced between the categories) and gender, i.e., a single different male appeared in each picture. Threat and neutral stimuli were also matched in terms of brightness, contrast, spatial frequency and complexity according to a previous study (Bradley et al., 2007). The pictures were purchased from Getty Images® (http://www.gettyimages.com), the International Affective Pictures System (IAPS, Lang et al., 2005) or produced by the authors with the support of a professional photographer. All of the pictures were similar in size (1024 × 768 pixels). Following the protocol developed by Lang et al. (1997), the pictures were rated on a scale of 1–9 in terms of pleasure and arousal by a separate group of 134 participants (104 female, 21.5 years ± 3.36) using the paper-and-pencil version of the Self-Assessment Manikin (Bradley and Lang, 1994). The means values of valence and arousal for each picture category are shown in Table 1.

### Experimental design

The stimuli were projected onto a screen located in front the participants and were viewed inside the scanner using a mirror attached to the head coil. Stimuli were presented using presentation software (Neurobehavioral Systems, version 11.0, Inc., Albany, CA, USA). In the beginning of each trial, the participants were instructed to attend to each picture while maintaining eyes fixed on a spot at the center of the screen. After 3 s of attending to the picture, a square appeared around the fixation spot 700–1200 ms prior to target onset. The target was a small annulus that appeared around the fixation spot. The picture, the square and the target remained on until the end of the trial, which had a total duration of 5 s. Subjects were required to press a button with the right index finger as quickly as possible after target onset. An MR-compatible response key, positioned on the right side of the participant's abdomen, recorded the responses. Each block consisted of 3 pictures (5 s each) of the same category (threat stimuli directed toward or directed away and neutral stimuli directed toward or directed away) presented in sequence, followed by 12 s of fixation cross. The experimental session consisted of 56 blocks (14 blocks of each category), pseudo randomized through the experiment and divided into 4 runs.

### Data pre-processing and general linear model analysis

The Statistical Parametric Mapping software package (SPM8, Wellcome Department of Cognitive Neurology, London, UK) was used for pre-processing and GLM analysis. The first 3 functional volumes of each run were removed to eliminate non-equilibrium effects of magnetization. The remaining images were corrected for head movement by realigning all of the images to the first image via rigid body transformations. The data were realigned to remove residual motion effects. For each participant, functional and structural images were co-registered. Structural data were segmented and normalized by matching them to the standardized MNI template (Montreal Neurologic Institute, Evans et al., 1993), and the transformation parameters estimated in this step were applied to all of the functional images. Finally, the functional images were spatially smoothed with an 8-mm Gaussian filter (FWHM).

GLM analysis was performed according to the framework implemented in SPM8 (Friston et al., 1995). For each participant a GLM model was built with the 4 experimental conditions entered in the design matrix as separate regressors. The 15 s experimental blocks were used as regressors of interest for each condition, and the 12 s fixation cross between the blocks served as a baseline. The regressors of interest were convolved with a canonical hemodynamic response function. Movement parameters from the realignment step were entered as covariates of no interest to control for participant's movement. The low frequency components were modeled by a set of discrete cosine functions (cut off period 128 s). Because the regressor for each condition included a period of picture observation and a period of task execution, the pattern of activation for each condition should capture both the affective and the task components. The 4 runs were first modeled independently; then, to investigate the emotional component in each context, contrast images were created between each threat condition (*directed toward* and *directed away*) versus their respective neutral conditions using all 4 runs. The contrast images represented the patterns of brain activation in response to *threat stimuli directed toward the viewer* and *threat stimuli directed away from the viewer*, discounting non-emotional effects. These contrast images were used as input to the pattern regression analysis.

### Pattern recognition analysis

**Fig. 1:**
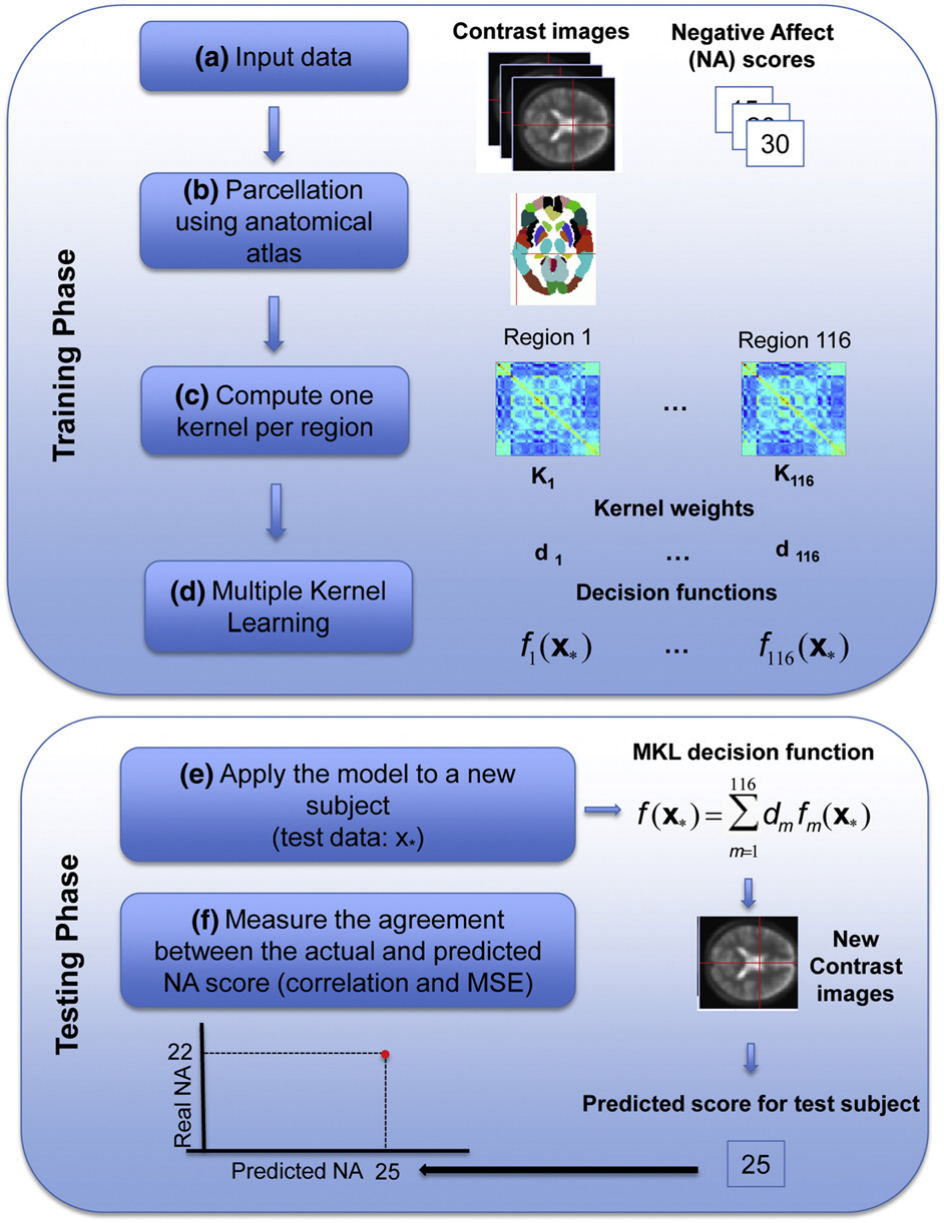
Multiple kernel learning frameworks. Superior panel: (A) The multiple kernel learning (MKL) regression model is trained by providing examples that pair a contrast image from the GLM model and a personality trait score. (B) The MKL framework uses a predefined anatomical template to segment the contrast images into 116 anatomical brain regions. (C) A linear kernel is computed for each brain region. (D) The MKL simultaneously learns and combines different models represented by different kernels or decision functions, i.e., the model learns the contribution of each region to the decision function (kernel weights), and within each region, the contribution of each voxel (voxel weights). Inferior Panel: (E) during the test phase, given the contrast image of a test subject the MKL model predicts the personality score. (F) The model performance is evaluated using 2 metrics to measure the agreement between the predicted and the actual negative affect scores, that is, Pearson's correlation coefficient (r) and the mean squared error (MSE). (Single column fitting image).

Pattern recognition analysis was performed according to the framework implemented in PRoNTo (Schrouff et al., 2013). We used pattern regression analysis to investigate the relationship between patterns of brain activity to threat stimuli (*directed toward* and *directed away*) and the positive and negative affect traits, i.e., we trained and tested one MKL regression model per threat stimulus (*directed toward* and *directed away*) and per score (NA and PA). The procedure for building pattern regression models consists of two phases: training and testing. During the training phase, the model is trained by providing examples that pair a spatial pattern (e.g., pattern of brain activity during an experimental condition) and a label (e.g., personality traits, psychological measures or clinical score). Once the model is “learned” from the training data (i.e., the model parameters are estimated based on the training data), it can be used to predict the label of a new test example. Here we used a pattern regression model that accounts for our knowledge about brain anatomy during the model estimation. This approach relies on Multiple Kernel Learning (MKL, Schrouff et al., 2014), which aims at simultaneously learning and combining different models, represented by different kernels. In the present case, each brain region corresponds to a different kernel. More specifically, the model will “learn” the contribution of each region for the decision function (kernel weights) and within each region the contribution of each voxel (voxel weights). This approach therefore corresponds to a hierarchical model, in which the models from each individual brain region are assembled to form the whole brain model. Since the MKL model currently implemented in PRoNTo assumes sparsity in the kernel combination (SimpleMKL, Rakotomamonjy et al., 2008), it will only select a subset of the regions to perform the regression (the remaining regions will have kernel weights equal to zero). We used a predefined anatomical template (Automated Anatomical Labeling, AAL template, Tzourio-Mazoyer et al., 2002) to delimitate the anatomical regions in the brain. The AAL template splits the brain into 116 anatomical regions. For each region a linear kernel was computed based on the regional pattern of activation containing all voxels within the region. The kernels were normalized (to compensate for the fact that the number of voxels varies among brain regions) and mean centered using standard operations in PRoNTo. Fig. 1 represents an overview of the pattern regression analysis based on MKL.

#### Model performance

The model performance was evaluated using 2 metrics to measure the agreement between the predicted and the actual scores, Pearson's correlation coefficient (r) and mean squared error (MSE). The correlation coefficient describes the strength of a linear relationship between 2 variables. A small correlation is an indication of poor predictions. The MSE is the mean of the squared differences between the predicted and true scores, it represents the mean error between the predicted and actual scores and is commonly used to evaluate the performance of predictive models.

We used a nested cross-validation procedure to train the model and optimize the model's hyperparameters. The external loop was used for assessing the model's performance (r and MSE) and the internal loop was used for optimizing the models hyperparameters (soft-margin parameter C for the SimpleMKL) using MSE as optimization criterion. We used a “leave-one-subject-out” cross-validation scheme for the external and the internal loop.

For all of the metrics statistical significance was determined using permutation tests, i.e., the same cross-validation procedure described above was performed 100 times with the labels permuted across the participants. The p-value was calculated by counting how many times the absolute value of the metric (r or MSE) with the permuted labels was equal or higher (or lower in case of the MSE) than the value obtained with the correct labels, and then dividing this number by 100. The results were considered significant if p-value < 0.05.

#### Model interpretation

As previously explained, the MKL model has 2 sets of weights, the kernel weights and the voxel weights. The kernel weights represent the contribution of each region and the voxel weights represent the contribution of each voxel within the regions for the decision function or predictive model. Both sets of weights can be explicitly computed and plotted as brain images to enable interpretation in terms of anatomically defined brain regions.

## Results

### Positive and negative affect scores

In the present study, we used a 2 sample t-test to assess possible gender differences on the scores. Neither the PA nor the NA scores differ between males and females t(32) = 0.715 p = 0.48 and t(32) = − 1.387 p = 0.17, respectively. The mean and standard deviations for the PA and NA scores for each gender and for all of the participants are present in Table 2.

### Pattern regression analysis

**Fig. 2:**
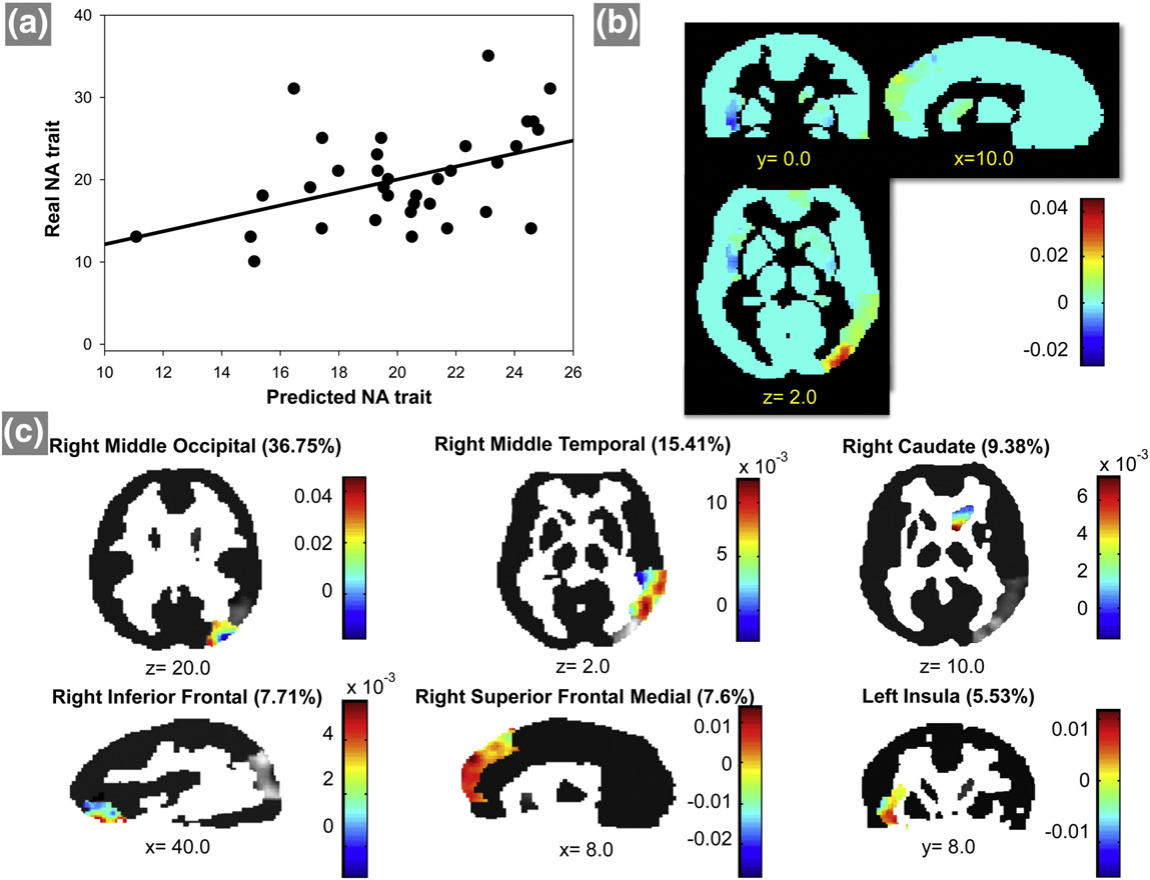
Multiple kernel learning (MKL) results. (A) Scatter plot between the real and predicted NA scores for the model based on patterns of brain activation to threat stimuli in the *directed away* context. The correlation and the MSE between the real and predicted NA scores were r = 0.52 (p-value = 0.01) and MSE = 24.43 (p-value = 0.01). (B) Weights per voxel in a whole brain fashion, the color bar represents the full range of the weights. The images show the coronal, sagittal and axial slices (MNI coordinates). (C) Top 6 regions ranked by the MKL model that were relevant to the prediction. The percentage of weight of each region are in parentheses. The colors represent the weights per voxel within each region, including the right middle occipital gyrus, right middle temporal gyrus, right caudate nucleus, right inferior frontal gyrus (orbital part), right medial frontal gyrus and left insula. (2-column fitting image).

Performance of the different models measuring the agreement between predicted and actual scores (PA and NA) based on patterns of brain activation in response to the different threat stimuli (*directed toward* and *directed away*) are presented in Table 3. The correlation coefficient (r) and MSE between the predicted and actual NA scores were significant for the model based on patterns of brain activation to threat stimuli in the *directed away* context. No significant results were found for the model based on patterns of brain activation to threat stimuli in the *directed toward* context. These findings indicate that the MKL model could decode NA scores from patterns of brain activation in response to the emotional component of threat stimuli in the *directed away* context, but not in the *directed toward* context. Fig. 2a shows the scatter plot between predicted and actual NA scores. No significant results were found for the MKL models trained to decode PA scores from patterns of brain activation during both threat stimuli.

Figs. 2b and 2c show the brain maps with the whole brain voxel weights and the 6 top regions ranked according to the region weights for the MKL model trained to decode NA based on patterns of brain activation to threat stimuli *directed away* from the viewer. In Figs. 2b and 2c, a positive value for the weight at a voxel means that as the brain activation increases at that voxel, so does the predicted scale, assuming that the brain activations at the remaining voxels are held fixed. On the other hand, a negative value for the weight means that the predicted scale reduces as the brain activation increases at that voxel, assuming that the brain activations at the remaining voxels are held fixed.

We emphasize that weight maps should not be interpreted as statistical parametric maps; they provide a spatial representation of the predictive function.

Table 4 shows the regions selected by the MKL model, which are ranked according to their contribution to the model (kernel or region weights). A total of 37 regions had a non-null contribution to the model.

## Discussion

The main goal of the present study was to apply pattern regression analysis to functional neuroimaging data to determine whether patterns of whole-brain activity to threat stimuli could be used to decode individual PA and NA traits, evaluated by the PANAS scale in a healthy sample. We applied a multiple kernel learning approach, considering the whole brain hierarchically as a combination of regional multivariate patterns (Schrouff et al., 2014). This approach enables us to identify a set of brain regions that are more informative about the variable being decoded. Although we failed to predict the PA trait, our results indicated that it is possible to decode the NA trait from patterns of brain activity in response to threat stimuli *directed away* from the viewer. These results suggest that pattern regression analysis can be effective for decoding measures of individual personality traits from patterns of brain activation to emotional stimuli.

The MKL approach identified 37 brain regions that were relevant to the predictive model. The first 12 most relevant regions, ranked according to their weights, comprised 95.5% of the total weights. Most of these regions (20 out of 37 regions) were in the right hemisphere (see Table 4). The right hemisphere has been proposed to have an important role in emotional processing and attention (Lane et al., 1999), particularly with respect to NA (Davidson and Irwin, 1999, Rohr et al., 2013, Whittle et al., 2006). Davidson and Irwin (1999) showed a positive correlation between the activation of the right amygdala and the NA trait in a sample of depressed patients, while other studies showed an association between NA and a network of regions in the right hemisphere (Rohr et al., 2013, Whittle et al., 2006).

Interestingly, the brain regions with the highest contribution for decoding the NA trait from patterns of brain activity in response to threat stimuli *directed away* from viewers were occipito-temporal regions, prefrontal structures (i.e., orbitofrontal gyrus, dorsal and ventral medial prefrontal cortex - dmPFC and vmPFC), insula, hippocampus and the cerebellum. These regions include important areas for emotion processing (Davidson and Irwin, 1999), threat stimuli perception (Kret et al., 2011) and negative affect processing (Rohr et al., 2013, Zhang et al., 2015). The occipital–temporal regions presented the highest contribution to the model with 56.1% of the total weights. Similar regions were found in a recent meta-analysis from Servaas et al. (2013) where they showed that activity in the middle temporal gyrus and middle occipital gyrus were correlated with the NA (neuroticism) trait in studies that investigated the processing of negative emotional versus neutral stimuli. In addition, occipital–temporal regions (such as the extrastriate body area) play an important role in the observation and execution of an action in emotional context (Downing et al., 2001, Ferri et al., 2013).

The prefrontal regions (i.e., the orbitofrontal gyrus, dmPFC and vmPFC) also had an important contribution to the predictive model, showing 16.1% of the total weights. Previous studies from Mobbs et al., 2007, Mobbs et al., 2009, Mobbs et al., 2010 suggested that the activation of prefrontal regions is related to the risk assessment during threatening conditions. These studies demonstrated that when the threat is more distant, higher-order cortical systems (such as the prefrontal cortex) are more active to provide better evaluation strategies to avoid the danger. In the present study, prefrontal regions were important to the predictive model based on patterns of brain activity in response to threat stimuli *directed away* from the viewer. One possible explanation for the contribution of prefrontal regions is that the pattern of activity in these regions varied greatly between subjects modulated by the level of NA of the subject. It is reasonable to suppose that the NA level modulates the risk evaluation in the *directed-away* threat condition, resulting in different patterns of activation in the prefrontal regions across subjects.

In the present study, the insula was another important emotional brain region contributing to the predictive model. Activity of this region has been implicated in interoceptive processes and affective information, contributing to emotional subjective experience (Craig, 2002, Wiens, 2005, Zaki et al., 2012). Interestingly, Mériau et al. (2009) have shown that insula activation is associated with individual differences (NA state) during visualization of emotional stimuli. More specifically, a higher NA state was associated with increased activity in the left insula.

Taken together, several emotional regions strongly contributed to the prediction of the NA trait from patterns of brain activity to threat stimuli. In the present study, we used threat versus neutral contrast in 2 contexts, 1 *directed toward* the viewer and 2 *directed away* from the viewer. The MKL model could only predict the NA trait in the context with the threat *directed away* from the viewer. One possible explanation for this finding is that the *directed-away* stimuli are more ambiguous, and people with different NA might react functionally, as measured by fMRI, in different ways to ambiguous threats (influenced by how they perceive the threat) but not to unambiguous ones. Accordingly, other studies demonstrated that individuals with higher NA trait (neuroticism) show heightened emotional reactivity (Haas et al., 2008) and experience more negative emotions (Clark et al., 1994). Following this rationale, the *directed-toward* stimuli probably evoked a more consistent pattern of brain responses across participants, resulting in the failure of the regression model to predict the NA trait. A previous study by Fernandes et al. (2013) showed that the threat directed toward the observer is considered as more intense, near, inescapable and with less possibility of hiding by the participants. Thus, it is likely that the increase in the magnitude of threat perception produced patterns of brain activities that were more homogeneous and less susceptible to individual variability.

Pattern recognition has been increasingly used in fMRI data analysis. Nevertheless, thus far, most applications of pattern recognition to neuroimaging data have focused on pattern classification analysis in which the model is trained to find discriminative patterns of brain activation between perceptual, cognitive, behavioral or clinical states. In these cases, the labels are assumed to be discrete values. More recently, pattern recognition techniques have also been used as regression models to identify relationships between patterns of brain structure or activity and continuous measures of individual variability (e.g., measures of personality trait or clinical scores). In this context, the labels are continuous instead of categorical values. An example of pattern regression analysis applied to fMRI in the neuroscience field is the study by Marquand et al. (2010), where ratings of subjective pain intensity to thermal stimuli were predicted from whole-brain patterns of activity in a healthy sample. Pattern regression has also been applied in other studies to answer theoretical questions about cognitive processes that could not be answered using traditional univariate methods (Cohen et al., 2010, Dosenbach et al., 2010, Kahnt et al., 2011). For example, Cohen et al. (2010) applied machine learning algorithms to patterns of brain activation during a response inhibition task to predict both subject's age and response time during the task. Dosenbach et al. (2010) applied support vector machine-based multivariate pattern analysis to predict individual's brain maturity across development from functional connectivity magnetic resonance imaging (fcMRI). Kahnt et al. (2011) applied similar approaches to investigate the role of different brain regions in multi-attribute decision, i.e., where the brain encodes the combined reward prediction and where it encodes the variability of the value predictions of the individual attributes. In the present study, the MKL model was applied to predict individual's NA and PA traits based on the pattern of brain activity in response to threat stimuli. These studies illustrate the great potential of using pattern regression models to predict measures of individual variability and decode complex cognitive and emotional processes.

In the clinical neuroscience field, pattern regression has been used to predict clinical measures, to identify biomarkers related to the severity of the disease and also to provide individualized, biological targets for treatment decisions (Borgwardt et al., 2013, Hoexter et al., 2013). Rizk-Jackson et al. (2011) applied pattern regression analysis to structural and functional MRI data to predict a quantitative measure of Huntington's disease progression on a sample of pre-Huntington's diseases patients. Furthermore, we have recently used pattern regression analysis to decode behavioral and emotional dysregulation symptoms from patterns of brain activation in youth (Portugal et al., 2016).

In the present study, we focused on the PA and NA traits as these are relatively stable personality characteristics over time (Watson, 1988a, Watson, 1988b). An interesting aspect of our findings is that the models were not able to predict PA from patterns of brain activity to threat stimuli. One possible explanation for these findings might be due to the fact that the PA scale is a very heterogeneous measure with different components (i.e., pleasantness and activation) that have distinct and sometimes even opposite courses (Egloff et al., 2003). In addition, it is possible that the large activation component (i.e., the extent to which one is feeling engaged or energized; Russell, 1980) of the PA scale could account for some unexpected results (Egloff et al., 1995, Kennedy-Moore et al., 1992). In spite of the failure to predict the PA trait, our results indicated that it is possible to decode the NA trait from patterns of brain activity to emotional stimuli. The NA trait can be considered a risk factor for the development of mental health disorders, especially affective disorders, such as anxiety and depression. Additional studies noted that NA can also be associated with poor prognosis (Clark et al., 1994) and can predict the onset of major depression (Ormel et al., 2004). Considering that high levels of NA can be considered as a risk factor for mental health disorders, decoding the NA trait from patterns of brain activity may be a potential biomarker of individual risk for future psychiatric illness.

There were some limitations in the present study. One limitation is the sample size; our results were obtained with a sample of 34 participants and therefore cannot be extrapolated as representative of large populations. Another limitation is the cross-validation framework. Although a *leave-one-out* cross-validation framework is commonly used in studies applying pattern recognition analysis to neuroimaging data, ideally the model should be trained and tested with truly independent samples. Further studies with larger sample sizes are needed to assess generalizability of these results by training and testing the model with completely independent samples. Finally, one limitation of the MKL regression model used in the present work is that the sparsity on the number of kernels (or regions in the present work) is imposed by an L1-norm regularization constraint, which does not select regions with correlated information. Future studies should explore MKL approaches, including a combination of L1 and L2 norms regularization constraints to address this limitation. A future direction to extend this work could apply pattern regression approaches to longitudinal follow-up studies to predict future outcomes and treatment response in patient populations.

## 6. Conclusion

In summary, our findings indicate that the MKL regression model has the potential to decode individual measures of personality from patterns of brain activation to emotional stimuli. Although we have failed to decode the PA trait, our results indicated that it is possible to decode the NA trait from patterns of brain activity to threat stimuli *directed away* from the viewer. The MKL approach allows investigating the contribution of different brain areas to predict a variable of interest (e.g., a personality trait or clinical score) by ranking and selecting the areas in an exploratory manner, i.e., not using a previous anatomical hypothesis. In the present study, the MKL model was able to identify a set of brain regions relevant to the prediction of the NA trait, including occipito-temporal regions, the orbitofrontal gyrus, dmPFC and vmPFC, and insula. These regions are related to threat perception, threatening context evaluation and emotional processing. Decoding the NA trait from patterns of brain activity to threat stimuli may be the first step toward identifying potential biomarkers of individual risk for future psychiatric disorders.

## Figures and Tables

**Table 1:** Mean values for pleasure, arousal, complexity ratings and physical features for each experimental condition.

	Valence	Arousal	Complexity	Brightness	Contrast	Spatial frequency
Directed toward threat	2.06 (1.22)	6.56 (2.20)	3.00 (0.74)	76.47 (23.75)	25.43 (9.73)	0.96 (0.10)
Directed toward neutral	5.20 (0.99)	3.65 (2.02)	2.92 (0.57)	79.33 (24.98)	27.67 (8.78)	0.99 (0.14)
Directed away threat	2.73 (1.37)	5.50 (2.16)	2.59 (0.77)	92.84 (36.86)	20.95 (10.57)	0.99 (0.06)
Directed away neutral	5.47 (1.01)	3.63 (1.99)	3.02 (1.04)	87.07 (37.10)	21.42 (7.37)	1.02 (0.11)

Note: Standard deviations are within parentheses. Brightness, contrast and spatial frequency were measure according to Bradley et al. (2007). The picture complexity was evaluated using a separate group of 58 participants (42 female, 20.5 years ± 1.88) on a scale of 1–9 in terms of figure-ground and complex scenes. Brightness was defined as the mean RGB (Red, Green and Blue) value for each pixel, averaged across all pixels for the pictures. Contrast was defined as the standard deviation of the mean RGB values computed across pixels for each column. Spatial frequency was defined as the median FFT (Fast Fourier Transform) power, which was computed for each row and column, and then averaged.

**Table 2:** Mean NA scores for all subjects and separated for men and women.

	All subjects	Men	Women
Positive affect	32.09 (5.12)	31.53 (5.23)	32.8 (5.06)
Negative affect	20.15 (5.86)	21.37 (6.58)	18.6 (4.55)

Note: Standard deviations are within parentheses.

**Table 3:** Performance model shown using correlation (r) and MSE between the real and predicted PA and NA scores for each threat context.

		r	p-Value	MSE	p-Value
Positive affect	Directed toward context	− 0.32	0.63	30.94	0.82
	Directed away context	− 0.23	0.42	29.67	0.73
Negative affect	Directed toward context	− 0.33	0.46	39.59	0.43
	Directed away context	0.52	0.01	24.43	0.01

Note: The p-value was obtained using a permutation test (100 permutations).

**Table 4:** Brain regions ranked according to their importance to the decision function for the model trained to predict NA scores from patterns of brain activation in response to threat stimuli *directed away* from the viewer.

Rank	Region label	Region weight (%)	Region size (voxel)
1	Occipital_Mid_R	36.75	1639
2	Temporal_Mid_R	15.41	3264
3	Caudate_R	9.38	953
4	Frontal_Inf_Orb_R	7.71	870
5	Frontal_Sup_Medial_R	7.60	1416
6	Insula_L	5.53	1866
7	Putamen_R	3.74	1062
8	Occipital_Inf_R	2.87	785
9	Cerebelum_Crus1_R	2.13	124
10	Rectus_L	1.99	101
11	Hippocampus_R	1.25	800
12	Parietal_Sup_L	1.12	1037
13	Olfactory_L	0.94	120
14	Temporal_Inf_R	0.75	1100
15	Vermis_1_2	0.50	47
16	Cerebelum_Crus2_L	0.35	3
17	ParaHippocampal_R	0.34	436
18	Frontal_Mid_Orb_L	0.28	333
19	Frontal_Mid_L	0.27	3236
20	Paracentral_Lobule_L	0.17	655
21	Calcarine_L	0.13	2062
22	Frontal_Inf_Oper_R	0.13	1200
23	Occipital_Mid_L	0.10	2850
24	Occipital_Sup_R	0.09	1079
25	Temporal_Pole_Mid_R	0.09	36
26	Parietal_Sup_R	0.08	736
27	Frontal_Inf_Tri_L	0.08	1969
28	Pallidum_L	0.07	270
29	SupraMarginal_R	0.04	1598
30	Frontal_Mid_Orb_L	0.04	406
31	Thalamus_R	0.02	1025
32	Supp_Motor_Area_R	0.02	1871
33	Cerebelum_4_5_R	0.02	562
34	Thalamus_L	0.01	1056
35	ParaHippocampal_L	0.00	370
36	Frontal_Mid_Orb_R	0.00	556
37	Cerebelum_6_L	0.00	1058

## References

[bb0005] Behroozi M., Daliri M.R. (2014). Predicting brain states associated with object categories from fMRI data. J. Integr. Neurosci..

[bb0010] Borgwardt S., Koutsouleris N., Aston J., Studerus E., Smieskova R., Riecher-Rössler A., Meisenzahl E.M. (2013). Distinguishing prodromal from first-episode psychosis using neuroanatomical single-subject pattern recognition. Schizophr. Bull..

[bb0015] Bradley M.M., Hamby S., Löw A., Lang P.J. (2007). Brain potentials in perception: picture complexity and emotional arousal. Psychophysiology.

[bb0020] Bradley M.M., Lang P.J. (1994). Measuring emotion: the Self-Assessment Manikin and the Semantic Differential. J. Behav. Ther. Exp. Psychiatry.

[bb0025] Britton J.C., Ho S.-H., Taylor S.F., Liberzon I. (2007). Neuroticism associated with neural activation patterns to positive stimuli. Psychiatry Res. Neuroimaging.

[bb0030] Calder A.J., Ewbank M., Passamonti L. (2011). Personality influences the neural responses to viewing facial expressions of emotion. Philos. Trans. R. Soc. Lond. Ser. B Biol. Sci..

[bb0035] Canli T. (2004). Functional brain mapping of extraversion and neuroticism: learning from individual differences in emotion processing. J. Pers..

[bb0040] Canli T., Sivers H., Whitfield S.L., Gotlib I.H., Gabrieli J.D.E. (2002). Amygdala response to happy faces as a function of extraversion. Science.

[bb0045] Canli T., Zhao Z., Desmond J.E., Kang E., Gross J., Gabrieli J.D.E. (2001). An fMRI study of personality influences on brain reactivity to emotional stimuli. Behav. Neurosci..

[bb0050] Carlson T.A. (2014). Orientation decoding in human visual cortex: new insights from an unbiased perspective. J. Neurosci..

[bb0055] Chadwick M.J., Hassabis D., Weiskopf N., Maguire E.A. (2010). Decoding individual episodic memory traces in the human hippocampus. Curr. Biol..

[bb0060] Clark L.A.A., Watson D. (1991). Tripartite model of anxiety and depression: psychometric evidence and taxonomic implications. J. Abnorm. Psychol..

[bb0065] Clark L.A.A., Watson D., Mineka S. (1994). Temperament, personality, and the mood and anxiety disorders. J. Abnorm. Psychol..

[bb0070] Cohen J.R., Asarnow R.F., Sabb F.W., Bilder R.M., Bookheimer S.Y., Knowlton B.J., Poldrack R.A. (2011). Decoding continuous variables from neuroimaging data: basic and clinical applications. Front. Neurosci..

[bb0075] Cohen J.R., Asarnow R.F., Sabb F.W., Bilder R.M., Bookheimer S.Y., Knowlton B.J., Poldrack R.A. (2010). Decoding developmental differences and individual variability in response inhibition through predictive analyses across individuals. Front. Hum. Neurosci..

[bb0080] Cohen M.X., Young J., Baek J.M., Kessler C., Ranganath C. (2005). Individual differences in extraversion and dopamine genetics predict neural reward responses. Cogn. Brain Res..

[bb0085] Costa P.T., McCrae R.R. (1992). Revised NEO Personality Inventory (NEO PI-R) and NEO Five-Factor Inventory (NEO-FFI).

[bb0090] Cox D.D., Savoy R.L. (2003). Functional magnetic resonance imaging (fMRI) “brain reading”: detecting and classifying distributed patterns of fMRI activity in human visual cortex. NeuroImage.

[bb0095] Craig A.D. (2002). How do you feel? Interoception: the sense of the physiological condition of the body. Nat. Rev. Neurosci..

[bb0100] Davidson R., Irwin W. (1999). The functional neuroanatomy of emotion and affective style. Trends Cogn. Sci..

[bb0105] Deckersbach T., Miller K.K., Klibanski A., Fischman A., Dougherty D.D., Blais M.A., Herzog D.B., Rauch S.L. (2006). Regional cerebral brain metabolism correlates of neuroticism and extraversion. Depress. Anxiety.

[bb0110] DeYoung C.G. (2010). Personality neuroscience and the biology of traits. Soc. Personal. Psychol. Compass.

[bb0115] Dosenbach N.U.F., Nardos B., Cohen A.L., Fair D.A., Power J.D., Church J.A., Nelson S.M., Wig G.S., Vogel A.C., Lessov-Schlaggar C.N., Barnes K.A., Dubis J.W., Feczko E., Coalson R.S., Pruett J.R., Barch D.M., Petersen S.E., Schlaggar B.L. (2010). Prediction of individual brain maturity using fMRI. Science.

[bb0120] Downing P.E., Jiang Y., Shuman M., Kanwisher N. (2001). A cortical area selective for visual processing of the human body. Science.

[bb0125] Egloff B., Schmukle S.C., Burns L.R., Kohlmann C.-W., Hock M. (2003). Facets of dynamic positive affect: differentiating joy, interest, and activation in the positive and negative affect schedule (PANAS). J. Pers. Soc. Psychol..

[bb0130] Egloff B., Tausch A., Kohlmann C.-W., Krohne H.W. (1995). Relationships between time of day, day of the week, and positive mood: exploring the role of the mood measure. Motiv. Emot..

[bb0135] Eisenberger N.I., Lieberman M.D., Satpute A.B. (2005). Personality from a controlled processing perspective: an fMRI study of neuroticism, extraversion, and self-consciousness. Cogn. Affect. Behav. Neurosci..

[bb0140] Eugène F., Lévesque J., Mensour B., Leroux J.-M., Beaudoin G., Bourgouin P., Beauregard M. (2003). The impact of individual differences on the neural circuitry underlying sadness. NeuroImage.

[bb0145] Evans A.C., Collins D.L., Mills S.R., Brown E.D., Kelly R.L., Peters T.M. (1993). 3D statistical neuroanatomical models from 305 MRI volumes. IEEE Conf. Rec. Nucl. Sci. Symp. Med. Imaging.

[bb0150] Ewbank M.P., Lawrence A.D., Passamonti L., Keane J., Peers P.V., Calder A.J. (2009). Anxiety predicts a differential neural response to attended and unattended facial signals of anger and fear. NeuroImage.

[bb0155] Fernandes O., Portugal L.C.L., Alves R.C.S., Campagnoli R.R., Mocaiber I., David I.P.A., Erthal F.C.S., Volchan E., de Oliveira L., Pereira M.G. (2013). How you perceive threat determines your behavior. Front. Hum. Neurosci..

[bb0160] Ferri S., Kolster H., Jastorff J., Orban G.A. (2013). The overlap of the EBA and the MT/V5 cluster. NeuroImage.

[bb0165] Friston K.J., Frith C.D., Turner R., Frackowiak R.S.J. (1995). Characterizing evoked hemodynamics with fMRI. NeuroImage.

[bb0170] Haas B.W., Constable R.T., Canli T. (2008). Stop the sadness: neuroticism is associated with sustained medial prefrontal cortex response to emotional facial expressions. NeuroImage.

[bb0175] Haas B.W., Omura K., Amin Z., Constable R.T., Canli T. (2006). Functional connectivity with the anterior cingulate is associated with extraversion during the emotional Stroop task. Soc. Neurosci..

[bb0180] Hamann S., Canli T. (2004). Individual differences in emotion processing. Curr. Opin. Neurobiol..

[bb0185] Harry B., Williams M.A., Davis C., Kim J. (2013). Emotional expressions evoke a differential response in the fusiform face area. Front. Hum. Neurosci..

[bb0190] Haynes J.-D., Rees G. (2005). Predicting the orientation of invisible stimuli from activity in human primary visual cortex. Nat. Neurosci..

[bb0195] Haynes J.-D., Sakai K., Rees G., Gilbert S., Frith C., Passingham R.E. (2007). Reading hidden intentions in the human brain. Curr. Biol..

[bb0200] He Q., Xue G., Chen C., Chen C., Lu Z.-L., Dong Q. (2013). Decoding the neuroanatomical basis of reading ability: a multivoxel morphometric study. J. Neurosci..

[bb0205] Hoexter M.Q., Miguel E.C., Diniz J.B., Shavitt R.G., Busatto G.F., Sato J.R. (2013). Predicting obsessive–compulsive disorder severity combining neuroimaging and machine learning methods. J. Affect. Disord..

[bb0210] Hooker C.I., Verosky S.C., Miyakawa A., Knight R.T., D'Esposito M. (2008). The influence of personality on neural mechanisms of observational fear and reward learning. Neuropsychologia.

[bb0215] Kahnt T., Heinzle J., Park S.Q., Haynes J.-D. (2011). Decoding different roles for vmPFC and dlPFC in multi-attribute decision making. NeuroImage.

[bb0220] Kamitani Y., Tong F. (2005). Decoding the visual and subjective contents of the human brain. Nat. Neurosci..

[bb0225] Kennedy-Moore E., Greenberg M.A., Newman M.G., Stone A.A. (1992). The relationship between daily events and mood: the mood measure may matter. Motiv. Emot..

[bb0230] Kosinski M., Stillwell D., Graepel T. (2013). Private traits and attributes are predictable from digital records of human behavior. Proc. Natl. Acad. Sci. U. S. A..

[bb0235] Kotov R., Gamez W., Schmidt F., Watson D. (2010). Linking “big” personality traits to anxiety, depressive, and substance use disorders: a meta-analysis. Psychol. Bull..

[bb0240] Kret M.E., Pichon S., Grèzes J., de Gelder B. (2011). Similarities and differences in perceiving threat from dynamic faces and bodies. An fMRI study. NeuroImage.

[bb0245] Lane R., Chua P., Dolan R. (1999). Common effects of emotional valence, arousal and attention on neural activation during visual processing of pictures. Neuropsychologia.

[bb0250] Lang B., Cuthbert L.,.P.J., Bradley M.M., Cuthbert B.N. (1997). Motivated attention: affect, activation, and action. Attention and Orienting: Sensory and Motivational Processes.

[bb0255] Lang P.J., Bradley M.M., Cuthbert B.N. (2005). IAPS: affective ratings of pictures and instruction manual. Emotion.

[bb0260] Markon K.E., Krueger R.F., Watson D. (2005). Delineating the structure of normal and abnormal personality: an integrative hierarchical approach. J. Pers. Soc. Psychol..

[bb0265] Marquand A., Howard M., Brammer M., Chu C., Coen S., Mourão-Miranda J. (2010). Quantitative prediction of subjective pain intensity from whole-brain fMRI data using Gaussian processes. NeuroImage.

[bb0270] Mériau K., Wartenburger I., Kazzer P., Prehn K., Villringer A., van der Meer E., Heekeren H.R. (2009). Insular activity during passive viewing of aversive stimuli reflects individual differences in state negative affect. Brain Cogn..

[bb0275] Mobbs D., Marchant J.L., Hassabis D., Seymour B., Tan G., Gray M., Petrovic P., Dolan R.J., Frith C.D. (2009). From threat to fear: the neural organization of defensive fear systems in humans. J. Neurosci..

[bb0280] Mobbs D., Petrovic P., Marchant J.L., Hassabis D., Weiskopf N., Seymour B., Dolan R.J., Frith C.D. (2007). When fear is near: threat imminence elicits prefrontal–periaqueductal gray shifts in humans. Science.

[bb0285] Mobbs D., Yu R., Rowe J.B., Eich H., FeldmanHall O., Dalgleish T. (2010). Neural activity associated with monitoring the oscillating threat value of a tarantula. Proc. Natl. Acad. Sci..

[bb0290] Oliveira L.A.S., Oliveira L., Joffily M., Pereira-Junior P.P., Lang P.J., Pereira M.G., Figueira I., Volchan E. (2009). Autonomic reactions to mutilation pictures: positive affect facilitates safety signal processing. Psychophysiology.

[bb0295] Ormel J., Rosmalen J., Farmer A. (2004). Neuroticism: a non-informative marker of vulnerability to psychopathology. Soc. Psychiatry Psychiatr. Epidemiol..

[bb0300] Paulus M.P., Rogalsky C., Simmons A., Feinstein J.S., Stein M.B. (2003). Increased activation in the right insula during risk-taking decision making is related to harm avoidance and neuroticism. NeuroImage.

[bb0305] Polyn S.M., Natu V.S., Cohen J.D., Norman K.A. (2005). Category-specific cortical activity precedes retrieval during memory search. Science.

[bb7815] Portugal L.C.L., Rosa M.J., Rao A., Bebko G., Bertocci M.A., Hinze A.K., Bonar L., Almeida J.R.C., Perlman S.B., Versace A., Schirda C., Travis M., Gill M.K., Demeter C., Diwadkar V.A., Ciuffetelli G., Rodriguez E., Forbes E.E., Sunshine J.L., Holland S.K., Kowatch R.A., Birmaher B., Axelson D., Horwitz S.M., Arnold E.L., Fristad M.A., Youngstrom E.A., Findling R.L., Pereira M., Oliveira L., Phillips M.L., Mourao-Miranda J. (2016). Can emotional and behavioral dysregulation in youth be decoded from functional neuroimaging?. PLoS One.

[bb0310] Rakotomamonjy A., Bach F., Canu S., Grandvalet Y. (2008). SimpleMKL To cite this version: SimpleMKL. J. Mach. Learn. Res..

[bb0315] Rizk-Jackson A., Stoffers D., Sheldon S., Kuperman J., Dale A., Goldstein J., Corey-Bloom J., Poldrack R.A., Aron A.R. (2011). Evaluating imaging biomarkers for neurodegeneration in pre-symptomatic Huntington's disease using machine learning techniques. NeuroImage.

[bb0320] Rohr C.S., Okon-Singer H., Craddock R.C., Villringer A., Margulies D.S. (2013). Affect and the brain's functional organization: a resting-state connectivity approach. PLoS One.

[bb0325] Russell J.A. (1980). A circumplex model of affect. J. Pers. Soc. Psychol..

[bb0330] Sanchez T.A., Mocaiber I., Erthal F.S., Joffily M., Volchan E., Pereira M.G., de Araujo D.B., Oliveira L. (2015). Amygdala responses to unpleasant pictures are influenced by task demands and positive affect trait. Front. Hum. Neurosci..

[bb9000] Schrouff J., Monteiro J., Rosa M.J., Portugal L.C.L., Phillips C., Mourao-miranda J. (2014). Can we interpret linear kernel machine learning models using anatomically labelled regions? Poster presented at the 20th Annual Meeting of the Organization for Human Brain Mapping.

[bb0335] Schrouff J., Rosa M.J., Rondina J.M., Marquand A.F., Chu C., Ashburner J., Phillips C., Richiardi J., Mourão-Miranda J. (2013). PRoNTo: pattern recognition for neuroimaging toolbox. Neuroinformatics.

[bb0340] Servaas M.N., van der Velde J., Costafreda S.G., Horton P., Ormel J., Riese H., Aleman A. (2013). Neuroticism and the brain: a quantitative meta-analysis of neuroimaging studies investigating emotion processing. Neurosci. Biobehav. Rev..

[bb0345] Shinkareva S.V., Mason R.A., Malave V.L., Wang W., Mitchell T.M., Just M.A. (2008). Using FMRI brain activation to identify cognitive states associated with perception of tools and dwellings. PLoS One.

[bb0350] Spiridon M., Kanwisher N. (2002). How distributed is visual category information in human occipito-temporal cortex? An fMRI study. Neuron.

[bb0355] Steinmetz P.N., Cabrales E., Wilson M.S., Baker C.P., Thorp C.K., Smith K.A., Treiman D.M. (2011). Neurons in the human hippocampus and amygdala respond to both low- and high-level image properties. J. Neurophysiol..

[bb0360] Stonnington C.M., Chu C., Klöppel S., Jack C.R., Ashburner J., Frackowiak R.S.J. (2010). Predicting clinical scores from magnetic resonance scans in Alzheimer's disease. NeuroImage.

[bb0365] Tzourio-Mazoyer N., Landeau B., Papathanassiou D., Crivello F., Etard O., Delcroix N., Mazoyer B., Joliot M. (2002). Automated anatomical labeling of activations in SPM using a macroscopic anatomical parcellation of the MNI MRI single-subject brain. NeuroImage.

[bb0370] Vergun S., Deshpande A.S., Meier T.B., Song J., Tudorascu D.L., Nair V.A., Singh V., Biswal B.B., Meyerand M.E., Birn R.M., Prabhakaran V. (2013). Characterizing functional connectivity differences in aging adults using machine learning on resting state fMRI data. Front. Comput. Neurosci..

[bb0375] Watson D. (1988). Intraindividual and interindividual analyses of positive and negative affect: their relation to health complaints, perceived stress, and daily activities. J. Pers. Soc. Psychol..

[bb0380] Watson D. (1988). The vicissitudes of mood measurement: effects of varying descriptors, time frames, and response formats on measures of positive and negative affect. J. Pers. Soc. Psychol..

[bb0385] Watson D., Clark L. (1992). On traits and temperament: general and specific factors of emotional experience and their relation to the five-factor model. J. Pers..

[bb0390] Watson D., Clark L.a., Tellegen A. (1988). Development and validation of brief measures of positive and negative affect: the PANAS scales. J. Pers. Soc. Psychol..

[bb0395] Watson D., Clark L.A.A. (1984). Negative affectivity: the disposition to experience aversive emotional states. Psychol. Bull..

[bb0400] Watson D., Clark L.A., Harkness A.R. (1994). Structures of personality and their relevance to psychopathology. J. Abnorm. Psychol..

[bb0405] Watson D., Gamez W., Simms L. (2005). Basic dimensions of temperament and their relation to anxiety and depression: a symptom-based perspective. J. Res. Pers..

[bb0410] Watson D., Naragon-Gainey K. (2014). Personality, emotions, and the emotional disorders. Clin. Psychol. Sci..

[bb0415] Watson D., Pennebaker J.W. (1989). Health complaints, stress, and distress: exploring the central role of negative affectivity. Psychol. Rev..

[bb0420] Whittle S., Allen N.B., Lubman D.I., Yücel M. (2006). The neurobiological basis of temperament: towards a better understanding of psychopathology. Neurosci. Biobehav. Rev..

[bb0425] Wiens S. (2005). Interoception in emotional experience. Curr. Opin. Neurol..

[bb0430] Zaki J., Davis J.I., Ochsner K.N. (2012). Overlapping activity in anterior insula during interoception and emotional experience. NeuroImage.

[bb0435] Zhang W., Li H., Pan X. (2015). Positive and negative affective processing exhibit dissociable functional hubs during the viewing of affective pictures. Hum. Brain Mapp..

